# Halo-tolerant plant growth-promoting bacteria-mediated plant salt resistance and microbiome-based solutions for sustainable agriculture in saline soils

**DOI:** 10.1093/femsec/fiaf037

**Published:** 2025-04-07

**Authors:** Hui-Ping Li, Hong-Bin Ma, Jin-Lin Zhang

**Affiliations:** School of Forestry and Prataculture, Ningxia University, Yinchuan, Ningxia 750021, China; Ningxia Grassland and Animal Husbandry Engineering Technology Research Center, Ningxia 750021, China; Key Laboratory for Model Innovation in Forage Production Efficiency, Ministry of Agriculture and Rural Affairs, Yinchuan 750021, China; School of Forestry and Prataculture, Ningxia University, Yinchuan, Ningxia 750021, China; Ningxia Grassland and Animal Husbandry Engineering Technology Research Center, Ningxia 750021, China; Key Laboratory for Model Innovation in Forage Production Efficiency, Ministry of Agriculture and Rural Affairs, Yinchuan 750021, China; State Key Laboratory of Herbage Improvement and Grassland Agro-ecosystems, College of Pastoral Agriculture Science and Technology, Lanzhou University, Lanzhou 730000, China

**Keywords:** halo-tolerant plant growth promoting rhizobacteria (HT-PGPR), mechanisms of HT-PGPR stress responses, microbiome, salt stress

## Abstract

Soil salinization has been the major form of soil degradation under the dual influence of climate change and high-intensity human activities, threatening global agricultural sustainability and food security. High salt concentrations induce osmotic imbalance, ion stress, oxidative damage, and other hazards to plants, resulting in retarded growth, reduced biomass, and even total crop failure. Halo-tolerant plant growth promoting rhizobacteria (HT-PGPR), as a widely distributed group of beneficial soil microorganisms, are emerging as a valuable biological tool for mitigating the toxic effects of high salt concentrations and improve plant growth while remediating degraded saline soil. Here, the current status, harm, and treatment measures of global soil salinization are summarized. The mechanism of salt tolerance and growth promotion induced by HT-PGPR are reviewed. We highlight that advances in multiomics technologies are helpful for exploring the genetic and molecular mechanisms of microbiota centered on HT-PGPR to address the issue of plant losses in saline soil. Future research is urgently needed to comprehensively and robustly determine the interaction mechanism between the root microbiome centered on HT-PGPR and salt-stressed plants via advanced means to maximize the efficacy of HT-PGPR as a microbial agent.

## Teaser

Halo-tolerant plant growth promoting rhizobacteria (HT-PGPR) are a valuable biological tool for mitigating the toxic effects of high salt concentrations. And the microbiome centered on HT-PGPR is solutions for sustainable agriculture in saline soils.

## Introduction

Soil salinization is one of the major factors threatening food security and ecosystem health in arid and semi-arid regions of the globe (Nachshon 2018). Currently, saline soil occupies ∼6% of the global land area (∼800 million hectares) and is a reserve land resource with important strategic significance (Lu and Fricke 2023). Unfortunately, soil salinization is accelerated via irrational use and exploitation of land. According to predictions, ∼50% of arable land area worldwide will be salinized by the middle of the 21st century (Yuan et al. 2022). Additionally, the United Nations Food and Agriculture Organization projects that a 50% increase in food production is required to meet the needs of a growing global population by the year 2050 (Shimoyama et al. 2020). If these saline soils can be maximized for agricultural production, the problems of the demand for food caused by the expanding population and the growth of spendable income can be mitigated. Hence, the improvement and utilization of saline soil have been highly valued.

The existence of excessive salt in soil exhibits cumulative and far-reaching effects on plants (Artiola et al. 2019). The water potential of the soil around the plant root rapidly decreases under salt stress, resulting in continuous osmotic stress that severely inhibits the growth of new leaves. Subsequently, high concentration of salts will trigger excessive accumulation of Na^+^ in the plant, causing ion imbalance, then ion toxicity and nutrient deficiency. Persistent high concentrations of salt will further lead to oxidative stress in plants, alter cell membrane permeability, and disrupt physiological and biochemical metabolism, ultimately altering plant growth and morphological development (Nabti et al. 2015, Mekawy et al. 2024). Faced with these challenges, on the one hand, plants have developed several pathways to cope with high salt stress, including salt tolerance-related transporters or channels, stress-sensing regulatory genes and proteins, on the other hand, external use of physical methods (flushing, scraping, leaching, etc.) and chemical amendments (addition of gypsum and lime). But, these methods have achieved only a bit success and limited practical significance (Egamberdieva et al. 2019). In addition, several other methods, such as modifying breeding practices, changing crop calendars, and introducing genetically engineered salt-tolerant plant varieties, have been evaluated for improving crop productivity in salt-affected soils. But, these methods have so far achieved only moderate success, because they are costly, time-consuming, and most importantly, lead to genetic erosion of indigenous species (Anderson et al. 2019, Arora et al. 2020a).

Halophilic microorganisms have the potential to remove the salt from saline soil, providing a unique prototype for studying the stress resistance, adaptation, and response processes of microorganisms, which may be integrated into crops to cope with various abiotic stresses (Chen et al. 2018, Khan et al. 2021). Among others, using halo-tolerant plant growth promoting rhizobacteria (HT-PGPR) to alleviate salt stress in plants via various physiological and molecular mechanisms is a natural restorative strategy to change plant salt tolerance (Alishahi et al. 2020, Etesami and Glick 2020, Najafi Zilaie et al. 2022). This method is preferred where it has been proved to be difficult to increase salt-resistant germplasm. Despite its numerous benefits, the precise processes of HT-PGPR help plants remain unclear. Therefore, researchers have been engaged in exploring the underlying mechanisms of HT-PGPR that stimulate plant growth and regulate salt tolerance, which has guiding significance for agricultural production and ecosystem management in saline lands.

## HT-PGPR-mediated salt tolerance in plants

**Figure 1.**
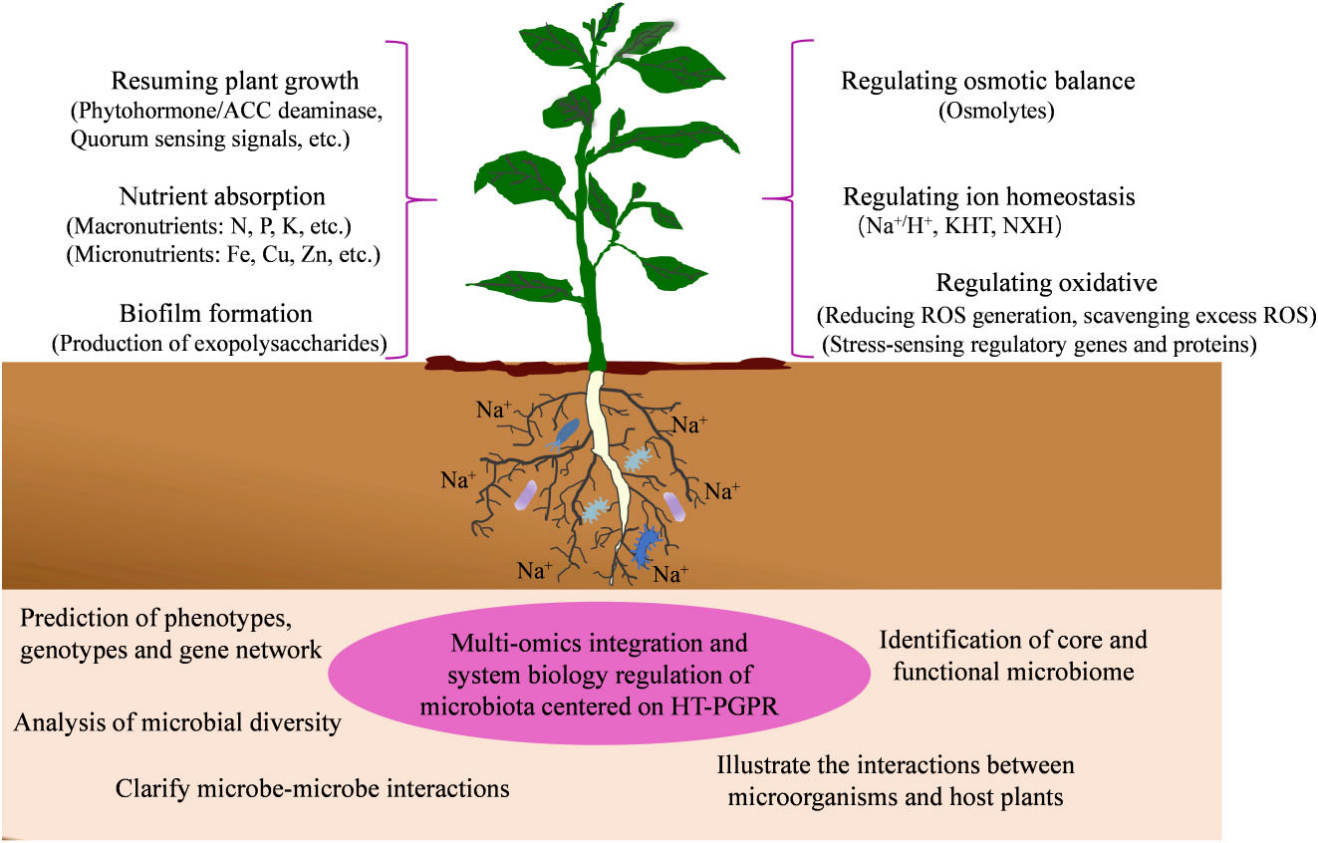
Mechanism of plant growth and salt tolerance mediated by HT-PGPR.

The salt tolerance of microbes far exceeds that of plants. HT-PGPR are a class of typical PGPR with unique genetic and inherent metabolic characteristics, which directly contribute to reducing the negative effects of salt stress on plants. However, the fascinating interactions that occur between HT-PGPR and stressed plants remain to be further investigated. Presumably, alleviation of salt stress via HT-PGPR can touch upon a three-layer interwoven action and connection: first, the survival of bacteria in the hyperosmotic conditions, second, the induction of salt tolerance events in plants, and finally, the amelioration of soil quality via diverse mechanisms (Saghafi et al. 2019, Alhindi and Albdaiwi 2022). The mechanism of HT-PGPR in alleviating salt stress in plants is depicted in Fig. 1. Recent work has also corroborated that HT-PGPR can regulate the expression of multiple genes involved in the amelioration of salt stress in plants (Table 1). Table 2 displays the inoculation effect of some PGPR strains on plant growth and salt tolerance.

**Table 1.** Expression of genes found in plants accountable for reducing the effects of salinity stress mediated by PGPR.

Strain	Plant	Modification of gene expression related to salt stress	Gene responsible for traits	Reference
*Alcaligenes faecalis* JBCS1294	*Arabidopsis thaliana*	Upregulated the expression of *AVP1, SOS1, HKT1*, and *CPD* in the shoots, but downregulated expression of *ERF* and *NHX1*	Ion transporters (*AVP1, SOS1, HKT1*, and *NHX1*), ethylene response factor (*ERF*), brassinosteroid biosynthetic cytochrome P450 (*CPD*), which regulated plant salt tolerance	Bhattacharyya and Lee (2017)
*Bacillus fortis* SSB21	*Capsicum annum* L.	Upregulated the expression profiles of *CAPIP2, CaKR1, CaOSM1*, and *CAChi2* genes	Stress-related genes, encodes plasma membrane intrinsic protein related in transportation of smaller neutral solutes and water	Yasin et al. (2018)
*Azospirillum lipoferum* FK1	Chickpea (*Cicer arietinum* L.)	Induced the expression of CAT, APX, SOD, PAL, PPO, CHS, CHI, DREB2A, and IFS-related genes.	Antioxidant enzymes and salt tolerance-related genes	El-Esawi et al. (2019)
*Bacillus megaterium* A12 (BMA12)	Tomato (*L. esculantum* cv. RioGrande)	Increased *Trxf, Trxm2*, and *Trxm1*/*2* gene expression levels, decreased *PsbA, PBGD*, and *LERBOH1* gene expression levels, positively influenced stress-related genes (*SOS1, APX1*)	Photosynthesis process (*PsbA, PBGD*), redox regulation (*Trxf, Trxm2, Trx m1*/*2*), and stress-related (*SOS1, APX1, LERBOH1*) gene	Akram et al. (2019)
*Arthrobacter woluwensis, Microbacterium oxydans, Arthrobacter aurescens, Bacillus megaterium*, and *Bacillus aryabhattai*	Soybean cultivar Pungsannamul	Stimulated the expression of *GmST1* and *GmLAX3*	Salt stress-responsive genes, which are greatly involved in ABA signaling and mitigating ROS stress	Khan et al. (2019b)
*Glutamicibacter* sp. YD01	*Oryza sativa* L.	*OsNHX1, OsHKT1, OsPOX1, OsFeSOD, OsGR2, OsDREB2A*, and *OsWRKY11* genes were upregulated	Ion transporters, antioxidant enzymes, transcription factor (ERF)-related genes responsive to salt stress and ethylene	Ji et al. (2020)
*Enterobacter cloacae* PM23	*Zea mays* L.	Upregulated APX and SOD-related genes	Stress-related genes (APX and SOD) helped to mitigate salinity stress and improved plant growth	Ali et al. (2022b)
*Halospseudomonas pachastrellae* GRRB3	Wheat cultivar (Akbar-19; Lot No. KL-690601)	Upregulated *WDREB2* gene, whereas downregulated *DREB6* gene	*WDREB2* and *DREB6* genes exhibited a close association with osmotic stress conditions	Aizaz et al. (2023)
*Bacillus tequilensis* (UPMRB9), *Bacillus aryabhattai* (UPMRE6)	Rice (*Oryza sativa* L.)	13 proteins were upregulated, 5 proteins were downregulated. An increase in the expression of 8 upregulated and 2 downregulated proteins in protein synthesis.	Involved in the photosynthetic process, regulated rice salt stress tolerance	Chompa et al. (2024)

**Table 2.** Effect of inoculation with PGPR strains on plant growth and salt tolerance.

Strain	Source	Crop	Response	Reference
*Bacillus firmus* (SW5)	Wheat rhizospheric soil, Egypt	Soybean (*Glycine max* L.)	Improved biomass yield, nutrient uptake, chlorophyll synthesis, osmolytes levels, total phenolic and flavonoid contents, and antioxidant enzyme activities of NaCl-stressed soybean plants. But significantly reduced the IC50 values for both DPPH and β-carotene-linoleic acid assays, and alleviated the contents of H_2_O_2_ and MDA in salt-stressed plants.	El-Esawi et al. (2018)
*Bacillus* sp. (strains SR-2-1 and SR-2-1/1)	Rhizosphere of sorghum, Pakistan	Potato (*Solanum tuberosum* L.)	Consortium treatment enhanced auxin production and regulated antioxidant enzyme production, and K^+^, Ca^2+^, K^+^/Na^+^, and Ca^2+^/Na^+^ in plants in both normal and salt affected soils	Tahir et al. (2019)
*Curtobacterium albidum* SRV4	Rhizosphere of saline soils, India	Rice (*Oryza sativa* L.)	Plant growth parameters, photosynthetic efficiency, modulation of osmolytes and antioxidative enzymatic activities were improved.	Vimal et al. (2019)
*Pantoea alhagi* NX-11	Roots of sea rice, China	Rice (*Oryza sativa* L. ssp. *japonica* “Nipponbare”)	A 133% and 52.8% higher K^+^/Na^+^ ratio and proline content under salt stress, as well as upregulated expression of proline synthase, downregulated expression of proline dehydrogenase, and enhanced antioxidant enzyme activities	Sun et al. (2020)
*Burkholderia phytofirmans* PsJN	Surface-sterilized onion roots, Canada	*Chenopodium quinoa*	*Burkholderia phytofirmans* PsJN and biochar when applied together significantly enhanced plant growth, grain yield, and nutrient contents of quinoa. Enzymatic/nonenzymatic antioxidant activities were decreased by integrated treatment	Naveed et al. (2020)
*Bacillus spizizenii* FMH45	The Sfax solar saltern located in the center of eastern Tunisian coast	Radish (*Raphanus sativus* L.)	Presented an increase in seedling length, vigor index, and biomass, chlorophyll content, membrane integrity, and phenol peroxidase concentrations, significantly improved K^+/^Na^+^ and Ca^2+^/Na^+^ ratios, as well as reduced malondialdehyde and hydrogen peroxide levels under saline conditions	Masmoudi et al. (2021)
Microbiome inoculation	Root environment of *Populus deltoides* × *P. euramericana* “Nanlin895” (NL895), China	Salt sensitive poplar plantlets (*P. deltoides* × *P. euramericana* “Nanlin8950,” NL895)	Compared to control, 33.8%, 18.0%, and 29.9% of the aboveground biomass was increased under no-salt (NS), low-salt (LS), and high-salt (HS) inoculation, respectively. Rhizosphere microbial communities of all treatments were taxonomically and functionally different across multiple stages, and the variation extent was larger in bacterial than in fungal communities.	He et al. (2021)
*Bacillus safensis* AL, *Bacillus pumilus* HR, and *Zhihengliuella halotolerans* SB	Rhizosphere soil of *Atriplex lentiformis, Seidlitzia rosmarinus*, and *Halostachys belangeriana*, Iran	Wheat (*Triticum aestivum* L.)	Higher grain yields were observed in the Narin variety inoculated with *B. safensis* AL and in the Qods variety inoculated with *Z. halotolerans* SB at all salinity levels. At 160 mM NaCl level, bacterial strains caused an increase of 10%∼45% in dry weight, K^+^/Na^+^ ratio, P and Ca content, chlorophyll *a*, crude protein, and seed amylose and amylopectin content of the Narin variety compared to the Qods variety. These bacteria also increased the 2,2-diphenyl-1-picrylhydrazyl radical scavenging capacity by 21%, phenol content by 32%, and proline content by 6% in the Narin variety compared to the Qods variety.	Hajiabadi et al. (2022)
*Bacillus atrophaeus* (YL07), *Planococcus soli* (YL10)	Rhizosphere soil, China	Maize (*Zea mays* L.)	Improved maize growth performance, biomass yield, and antioxidant levels under salt stress. Protected maize from salt stress by regulating plant hormone [IAA and abscisic acid (ABA)] levels and increasing nutrient acquisition, increased the K^+^/Na^+^ ratio.	Hou et al. (2022)
*Agrobacterium tumefaciens* (B1), *Bacillus subtilis* (B2), and *Lysinibacillus fusiformis* (B3)	Root nodules of leguminous plants (common bean, Yardlong bean, Dhaincha, and Shame plant), Pakistan	Wheat (*Triticum aestivum* L.)	Strain B1, B2, and B3 dramatically boosted rice seedling development characteristics under salt stress conditions. Furthermore, B1 was shown to be a possible strain compared to other two bacteria that might aid in reducing salt stress and fostering plant development activities	Mahmud et al. (2023)
*Microbacterium azadirachtae* CNUC13	Rhizosphere soil samples of maize (*Zea mays* L.), South Korea	Maize (*Zea mays* L.)	Protected maize from salt stress by enhancing photosynthetic pigment synthesis (chlorophyll and carotenoids), reducing accumulation of osmotic (proline) and oxidative (ROS and MDA) stresses, and normalizing antioxidant enzymatic activities (catalase, SOD, and peroxidase)	Luo et al. (2024)

### Osmolyte accumulation

Osmotic adjustment is one of the essential characteristics of plant salt tolerance. When the salt ion concentration in the soil is very high, the osmotic potential of the soil is attenuated, making it difficult for plant roots to absorb water, resulting in osmotic stress (Egamberdieva et al. 2019). There are two ways for plants to regulate their permeability: one is to absorb and accumulate inorganic ions, such as Na^+^, K^+^, amd Cl^−^ in cells, and the other is to accumulate a certain amount of soluble organic substances to ensure their survival under high salt stress. Compatible solutes (CS) such as proline and sugar are universally known osmoprotectants that maintain cell expansion to prevent oxidative stress (da Cunha et al. 2023). Significantly, the homeostasis of proline is vital for sustaining growth under long-term stress. During stress, proline is accumulated, which can be degraded to provide supply energy and drive plant growth once the stress is relieved. Studies have also demonstrated that several CS, such as glycine betaine (GB)-like quaternary ammonium compounds, are not generated in arabidopsis and rice, etc. under nonsaline environments, but are significantly accumulated in plants under stress conditions (Radhakrishnan and Baek 2017). Furthermore, sugars (mannitol, trehalose, and fructose) accumulate in plants under salt stress, avoiding structural and functional changes in proteins and membranes. These results can be attributed to the most important property of CS in the context of salt resistance is their absolute compatibility with sensitive proteins and cell structures, even at high concentrations of CS that required to balance the osmolarity of the cell’s interior with high outside osmotic pressure by diverse salts, which are poised to cell functions.

It is noteworthy that microorganisms subjected to osmotic pressure in a saline environment accumulate large amounts of osmoprotectants in their cytoplasm (Ilangumaran and Smith 2017). Among them, HT-PGPR can synthesize osmotic substances with low molecular weight, electrically neutral and highly solubility, including soluble sugars, proline, amino acids, quaternary amines, and polyols, which can quickly scavenge reactive oxygen species (ROC) (Mishra et al. 2021). *Bacillus* sp. wp-6 was reported to influence the synthesis of cell wall and soluble sugar by regulating the expression of proteins (α-GAL, UGE1, UGE3, CWINV1, CWINV4, and UGP) in wheat seedlings under salt stress (Zhao et al. 2022). Inoculation of HT-PGPR strains *Enterobacter cloacae* HSNJ4 in canola (*Brassica napus* L.), the proline content in the treatment group was significantly increased by 61.4% and 47.2% compared with the control group, respectively, under 50 and 100 mM NaCl concentrations. The Malondialdehyde (MDA) content was significantly decreased by 14.4% and 19.6% than that in the control group, respectively (Li et al. 2017), indicating that HT-PGPR can reduce the loss of intracellular water by increasing osmotic adjustments, thereby mitigating osmotic stress and preserving osmotic balance in plant cells. And, the expression of some biosynthesis gene encoding CS, such as, *ectD*, the hydroxyectoine biosynthesis gene (Tao et al. 2016), and *GmOLPb*, osmotin-like protein b isoform gene (El-Esawi et al. 2018) were induced by HT-PGPR, making plants have a stronger ability to cope with osmotic stress.

### Ion homeostasis

The accumulation of Na^+^ in the plant cytoplasm is toxic to plant cells, as it can compete with other ions to bind proteins, causing the inactivation of enzymes. The regulation of Na^+^ uptake and transport in salt-stressed plants has been interpreted as maintaining a high K^+^/Na^+^ ratio in the cytoplasm. Many microbial strains have been shown to increase K^+^/Na^+^ ratios in plant shoot and/or root by increasing K^+^ and/or reducing Na^+^ in the cytoplasm, but the underlying mechanisms vary (Najafi Zilaie et al. 2022). The major aspect of salt stress tolerance in plants mediated by HT-PGPR involves the generation of responsive machinery to pool out the toxicity and establish an ion equilibrium state to avoid desiccation and flaccidity in plant cells. Among numerous salt tolerance-related transporters or channels, including the plasma membrane Na^+^/H^+^ antiporter (SOS1), Na^+^/H^+^ antiporter (NHX), K^+^ transporter 1 (AKT1), high-affinity K^+^ transporter (HKT), and K^+^ uptake transporter (KUP1), HKT1 plays crucial roles in Na^+^ influx across the plasma membrane, and long-distance transport of Na^+^ is considered a key determinant of plant salt tolerance (Etesami and Glick 2020). The specific role of HKT1 in HT-PGPR-mediated plant Na^+^ reduction under salt stress has been recognized (Hou et al. 2022). HT-PGPR also constrict the uptake of Na^+^ by altering the composition of the cell wall/cell membrane, which increases the electrogenic Na^+^/H^+^ ionic porters, while improving the expression of salt overly sensitive (*SOS*) genes and NHX transporters in plants. Niu et al. (2016) discovered that *Bacillus subtilis* (GB03) reduced Na^+^ transport from root to shoot to enhance the salt tolerance of *Puccinellia tenuiflora* by triggering upregulation of *PtHKT1;5* and *PtSOS1*, downregulation of *PtHKT2;1*. But, a study showed that *Bacillus megaterium* ZS-3 determines the downregulation of *HKT1* and the upregulation of *NHX1* and *AVP1* (a vacuolar H^+^-pyrophosphatase) pumping H^+^ into vesicles against Na^+^ toxicity (Shi et al. 2022). Furthermore, volatile organic compounds produced by *Bacillus amyloliquefaciens* FZB42 can induce the expression of genes (*NHX1*; Na^+^/H^+^ exchanger 1 and *HKT1*; high-affinity K^+^ transporter 1) to decrease the Na^+^ contents of the whole plants, thereby alleviating Na^+^ toxicity. Given the above, PGPR mainly involve in Na^+^ homeostasis and vacuolar compartmentation by mediating vacuolar membrane-bound NHX and HKT under salt stress, which can contribute to mitigate the effects of excessive accumulation of salt in plants.

### Oxidative stress response

A large amount of ROS is accumulated in plants under salt stress, and if they cannot be removed in time, the dynamic balance between the production and scavenge of ROS will be disturbed, causing peroxidation and degreasing of membrane lipid, destruction of membrane proteins and membrane lipids, and ultimately lead to programmed cell death (Xu et al. 2024). It has been extensively reported that HT-PGPR can reduce plant electrolyte leakage and lipid peroxidation, which are signatures of ROS-induced plasma membrane degradation (Liu et al. 2022). In addition to reducing plant ROS production, HT-PGPR can boost the ROS detoxification mechanism. When plants are exposed to salt stress, both the enzymatic antioxidant system, including superoxide dismutase (SOD), catalase (CAT), ascorbate peroxidase (APX), glutathione peroxidase (GPX), and the nonenzymatic system, including glutathione (GSH), ascorbic acid, and proline, are also activated. These signals stimulate plants to respond to high salt stress (Ali et al. 2022a). Yuan et al. (2020) discovered that *Pseudomonas*-produced phenazine reduced ROS accumulation while enhancing the activity of the antioxidant enzyme (catalase) in the leaves of seedlings cultivated in saline conditions. The crucial role of promoting plant ROS scavenging is confirmed by the fact that the salt tolerance induced by *B. amyloliquefaciens* SQR9, and some genes involved in glutamine synthetase and glutathione reductase were activated (Chen et al. 2017).

In contrast, several microbes with high antioxidant activity weakened the antioxidant system induced by salt stress. For example, inoculation of halo-tolerant *Brevibacterium linens* RS16 was reported to reduce the plant antioxidant enzyme activity, lipid peroxidation, and it also controlled the buildup of salt via modulating the activity of vacuolar H^+^ ATPase (Chatterjee et al. 2018). Rhizobacteria SUA-14 and SHM-13 selected on the basis of strong salt tolerance and excellent plant growth-promoting traits has reduced the CAT and SOD activity levels of maize in saline conditions compared to noninoculated maize (Shabaan et al. 2022). These studies suggest that HT-PGPR can also enhance the salt tolerance of plants by directly scavenging ROS, thereby reducing the production of stress-induced antioxidant in plants.

### Production of exopolysaccharides

Halo-tolerant bacteria tend to produce extracellular polymeric substances/exopolysaccharides (EPSs) in stressful environments to accelerate the formation of rhizosphere microbial biofilms, which can act as a defensive and protection mechanism against specific and nonspecific host immunity (Joulak et al. 2020, Ramasamy and Mahawar 2023). Under saline environment, EPSs produced by bacteria account for 40%–90% of their weight and can form a clay layer or capsule that tightly surrounds the bacterial cells, which can bind with ionic salts in the soil, and prevent these ions from reaching the stem, thereby improving the availability of nutrients and water from the rhizosphere, endowing plants with salt tolerance (Sun et al. 2020, Sunita et al. 2020, Chen et al. 2024). Previously, Mahmood et al. (2016) studied the role of EPS-producing halo-tolerant *Enterobacter cloacae* and *Bacillus drentensis* in improving the growth of salt-stressed mung bean by increasing the water uptake and nutrient availability. EPS production by HT-PGPR is also associated with water retention, nodulation, soil aggregation, humification, quorum sensing (QS), and the establishment of microbial diversity that protects plant cells from desiccation in a saline environment (Arora et al. 2020a). Furthermore, EPS-producing HT-PGPR possesses antioxidant properties that confer tolerance against salinity-induced oxidative damage (Sunita et al. 2020). Inoculation of *Pantoea alhagi* NX-11, an EPS-producing endophyte, alleviated salt stress damage and improved the growth of *Oryza sativa* by stimulating antioxidant activity (Sun et al. 2020). In another study, the combined effect of silicon dioxide (SiO_2_) nanoparticles and exopolysaccharide (EPS)-producing bacterial inoculum on the upregulation of antioxidant activities in *Solanum lycopersicum* under salinity stress had been reported (Kang et al. 2019). This suggests that EPS produced by HT-PGPR affects their physiology and adaptations to hostile conditions.

### ACC deaminase production

When plants are stressed by salinity, drought, and pathogens, excess ethylene is released, which seriously hinders root development (Qin et al. 2016). It is noteworthy that HT-PGPR exhibit the capability to produce 1-aminocyclopropane-1-carboxylic acid (ACC) deaminase, which catalyzes the conversion of ACC (the precursor of ethylene in all higher plants) to α-ketobutyrate and ammonia, thereby lowering ACC levels and preventing excessive increases in the synthesis of ethylene under various stress conditions, which is considered to be one of the effective mechanisms for inducing salt tolerance in plants (Orozco-Mosqueda et al. 2020, Gupta et al. 2021). Thus, plants with low ethylene levels will eventually overcome salt-induced growth suppression by interacting with ACC deaminase-producing bacteria. For example, Win et al. (2018) found that the expression of ACC deaminase in the endophytic *Pseudomonas* strain OFT5 mitigated the harmful effects of salinity on plant growth and physiological performance to some extent, which was manifest in a significant role in improving tomato plant growth, photosynthetic performance, and ion balance. Moreover, the *acd*S gene is a marker to determine whether HT-PGPR has the potential activity of ACC deaminase. Li et al. (2015) quickly and reliably assessed ACC deaminase activity in HT-PGPR by designing consensus-degenerate hybrid oligonucleotide primers (acdSf3, acdSr3, and acdSr4) with better performance than other primer sets. Undoubtedly, the production of ACC deaminase is one of the main mechanisms by which HT-PGPR fulfill the beneficial function in saline agroecosystems.

### Plant growth regulators

Phytohormones are small chemicals that play a key role in plant growth and development. Under saline conditions, phytohormone such as auxin, gibberellin (GA), cytokinin (CKs), and brassinosteroids (BRs), as well as other hormones including abscisic acid (ABA), ethylene, salicylic acid (SA) and jasmonic acid (JA), brassinosteroids (BRs), and pineolactones (SLs) are activated to regulate the synthesis, signal transduction, and metabolism of various hormones in plants to build defense systems. The yield loss in crops can be minimized by using phytohormone-producing HT-PGPR under salt stress. Kang et al. (2019) reported that a halo-tolerant PGPR *Leclercia adecarboxylata* MO1 could reprogram plants under salinity stress via Indole-3-acetic acid (IAA) production and ACC deaminase synthesis to significantly modulate plants’ endogenous sugar, organic acids, amino acids, and stress responsive ABA, thereby improving their growth and providing resistance. Apart from auxins other phytohormones (cytokinins, gibberellin, ABA, ethylene, etc.) have also been proved to play a role in alleviating the effect of salt stress in plants (Parray et al. 2016, Barnawal et al. 2017, Kumawat et al. 2023). A study found that a decrease in endogenous ABA and JA level of soybean plants inoculated with *Arthrobacter woluwensis* AK1 underelevated salt stress, and a similar ameliorative trend was observed for total proteins, polyphenol oxidase, and peroxidase activity under saline conditions, thus showing an enhanced stress mitigation (Khan et al. 2019a). This study also revealed that bacterial inoculation upregulated the expression of *GmLAXs* and *GmST* genes compared to uninoculated plants, which are involved in ABA-dependent pathway of plants exposed to salt stress (Khan et al. 2019b). A similar mechanism of tolerance has also been reported in herbaceous plants when *B. amyloliquefaciens* FZB42 conferred salt tolerance in *Arabidopsis* by upregulating plant JA/ethylene pathways (Liu et al. 2020). This again highlights the correlation between plant salt tolerance and beneficial microorganisms, as well as the mechanism at a molecular level against salt stress with the help of HT-PGPR.

### Nutrient acquisition

High electrolyte concentration and salt ion enrichment in saline soil enhance the adsorption of nutrient elements, such as P. Moreover, ion competition happens when salt and nutrient ions cross the cell membrane and are absorbed by plants, thus reducing the transformation of nutrient elements in soil and their migration to plants (Meinzer et al. 2023). For example, Na^+^ can compete with K^+^ for binding sites, and excessive Na^+^ leads to insufficient K^+^ for plants and nutritional imbalance. However, the nutrient mineralization and rhizosphere pH changes induced by HT-PGPR can increase the nutrient supply of plants. Under controlled and field conditions, the positive effects of inoculation with HT-PGPR on crop productivity in saline–alkali soil have been widely reported and have become the subject of many past reviews (Yasin et al. 2018, Egamberdieva et al. 2019, Saghafi et al. 2019, Chauhan et al. 2022, John et al. 2023). As Jiang et al. (2021) found that the combined application of halo-tolerant phosphate-solubilizing bacteria (PSB) *Providencia rettgeri* TPM23 and rock phosphate could significantly increase the plant length, biomass, and uptake of phosphorus (P) of peanut (*Arachis hypogaea* L.) through acidification, chelation, exchange reactions, release of complexing, or mineral dissolving compounds (e.g. organic acids), meanwhile improving the structure of the saline soil microbial communities.

But, considering the HT-PGPR-mediated nutrient acquisition of plants under salinity stress that is observed in the laboratory, several other studies have demonstrated the inconsistent effects of HT-PGPR under field conditions, with no clear links between the increase of plant nutrient absorption and the inoculation of HT-PGPR (Raymond et al. 2021, da Cunha et al. 2023). Consequently, it is necessary to explore how the observed effects under controlled conditions can be translated into consistent and positive effects in the field, in order to truly leverage the role of HT-PGPR.

## HT-PGPR as a soil remediation agent

Research has shown that HT-PGPR can improve nutrient status and soil structure of saline soils via modulating organic matter, pH, and ionic salt deposition, etc., thereby reviving the lost vegetative index (Zheng et al. 2019). Specifically, HT-PGPR mitigate ionic toxicity by cation bridging, hydrogen bonding, and anion adsorption (Arora et al. 2020a). There are reports that under high-salt stress conditions, bacterial Na^+^/Ca^2+^ transporters maintain intracellular acid–base balance and osmotic pressure stability by facilitating the transport of Na^+^ and Ca^2+^. This process leads to the accumulation of Ca^2+^ within bacterial cells while reducing soil Ca^2+^ content (Zhang et al. 2022). Arora et al. (2016) found that applying consortia of halophilic bacteria could enhance the biochemical properties of sodic soil by lowering the soil pH from 9.4 to 8.6, and significantly increasing microbial biomass C by 67.07% compared to control. And an increase in available P content of saline soil was observed by coupling PSB with rock phosphate (Adnan et al. 2020). A decrease in soil pH, electrical conductivity, and an increase in the accessibility of macronutrients (N, P, and K), enzyme activities (urease, alkaline phosphomonoesterases) and organic matter were reported upon inoculating saline soil with HT-PGPR and phosphogypsum (Jiang et al. 2021). In addition to balancing the stoichiometry of soil nutrients, HT-PGPR can bind to clay particles in the soil to form stable structural aggregates, increase soil permeability and aeration, thus regulating the diversity and spatial heterogeneity of soil matrix, which favor root system to capture nutrients and water (Siebers and Kruse 2019, Arora et al. 2020b). Thus, studies clearly support the role and potential application of HT-PGPR in enhancing soil quality affected by abiotic challenges like salinity.

## Implementation of omics technology in microbe-mediated plant salt tolerance

In recent years, advanced “-*omics*” technologies have enabled us to gain insights into the network of interactions between plant salt tolerance and beneficial microbes (Zheng et al. 2021, Wilbanks et al. 2022). Previous studies have predominantly focused on the binary interactions between plants and individual putatively mutualistic microbes (Yuan et al. 2016, Qin et al. 2018, Zhang et al. 2019). Unfortunately, when such microbes as single strains are introduced into soils, they sometime fali to improve plant growth and stress tolerance, which is largely due to competition with native soil microbial communities and poor colonization effectiveness. The rapid development of rhizosphere microbiome research has revived the belief that plants may benefit more from interactions with various microbial communities than from individual members of a community, which can help us gain a better understanding of how these microorganisms survive in saline soils and how they affect soil health and regulate ecosystem functions (Fig. 1). Here, we highlight that microbiota refers to the microbial community in a certain habitat, whereas the microbiome includes microbiota and their structural components (such as nucleic acids and proteins) and metabolites (Zheng et al. 2024). Li et al. (2024b) employed metagenomics, which revealed that plants can recruit the specific salt-tolerant bacterial consortium and associated functions instead of individual bacterial members for enhancing plant adaptability to salt stress. Another study demonstrated that the core microbiome in the rhizosphere of halophyte *Suaeda salsa* holds genes contributing to salt stress acclimatization, nutrient solubilization, and competitive root colonization in coastal saline soils (Yuan et al. 2016). Furthermore, the metabolic processes and gene regulation of living microbes under salt stress conditions can also be revealed by transcriptomics (Bajay et al. 2023). It is crucial to uncover the molecular mechanisms of salt stress on microbial and plant cells using these multiomics techniques, which also include metabolomics and metaproteomics, because multiomics integration analyses can reveal the connections and offer valuable insights into various biological factors responsible for important traits in stressed plants, so as to better utilize beneficial microorganisms to alleviate the detrimental effects of salinity in plants.

### Synthetic microbial communities

Synthetic microbial communities (SynComs) are created by artificially combining of two or more distinct cultured microorganisms with definite taxonomic status and specific functional traits, etc. in predetermined proportions under specified conditions (Li et al. 2024b). Compared with individual microorganisms, they exhibit reduced metabolic burden due to a division of labor, exchange resources, possess expanded metabolic capabilities, and constantly communicate (physically or chemically), resulting in high efficiency, strong functionality, good controllability, ease of preservation, and ease of application, thus better resisting environmental stress or invasions by other species (Jing et al. 2024). SynComs composed of PGPR with halophilic properties can enhance the plant defense against environmental stress, and increase productivity and sustainability through microbial interactions or microbial–plant interactions (Jing et al. 2024). A SynCom combining five bacterial strains, which were isolated from desert, could stimulate tomato plant growth under saline conditions and significantly increase the expression of salt stress‐related genes (Schmitz et al. 2022). When multiomics technologies are being widely applied in the fields of the root-associated microbiota of salt-stressed plant, some key analysis methods or techniques, such as PhyloChip (Berendsen et al. 2018) and single-cell Raman-D_2_O (Li et al. 2024a), can more sensitively and accurately analyze the differences and function of root microbial communities, and targeted sorting of microbial cells with defined functional properties in specific environments. These advancements will open a new avenue for capitalizing on the cultivable microbiome to strengthen plant salt tolerance, thus optimizing agricultural practices and production under saline conditions. The composition of microbial community will become unstable due to the growth competition among different strains, and the fluctuation of microbial population may decrease the efficiency of synthesizing the target products from microbial communities. Therefore, the challenge of building SynComs that can function in saline–alkali soils is to control the population and regulate the interaction between different strains.

### Bacterial and fungal symbionts

Some Sebacinales have associated bacteria, which recruit distinct microbes into their hyphosphere and thus together act effectively to perform a variety of functions, including increasing nutrient uptake, transport of essential nutrients responsible for plant productivity, enabling plants to survive under salt stress, high temperatures, waterlogging, and conferring resistance to toxins, heavy metal ions, and pathogenic organisms, as well as other beneficial effects such as enhance resistance tolerance against various abiotic stresses, promote plant growth, and enhance grain quality and alteration in the secondary metabolites (Varma et al. 2013, Bokati and Craven 2016). According to recent data, it has been demonstrated that SynComs formed of Sebacinales and PGPR members have been proven to possess functionality and universality (Moreno-Lora et al. 2023a, Jin et al. 2024, Maurice et al. 2024, Wang and Kuzyakov 2024). Nunes et al. (2022) discovered that the introduction of the plant growth-promoting microbial inoculants *Bacillus simplex* and *Penicillium bilaiae* altered the composition and possible functioning of the seed-associated bacterial communities of winter wheat. The dynamics of the phosphorus cycling genes (*ppt, ppx*, and *cphy*), utilization of organic phosphorus sources (*pho*D, *pho*X, and *phn*K), and the relative abundance of genes involved in organic nitrogen metabolism (*ure*C and *gdh*A), and ammonium oxidation (*amo*A) were increased. Another study revealed that the composition and multifunctionality of cultivable fungal and bacterial communities were driven by salinity levels, where plant recruited microbes from the rhizosphere and soil in closest proximity to the roots (Kimbrough et al. 2019). Additionally, there is evidence on the multifunctionality of bacterial and fungal symbionts in shaping the global plant microbiota network via a comprehensive co-occurrence network analysis, including rhizosphere and root samples from six plant species in a natural desert (Maurice et al. 2024). Therefore, coordination is a vital feature of utilizing different microorganisms, but further assessment is still needed on how they persist and how they affect microbial function and resilience of the global plant microbiome under different environmental conditions.

### Quorum sensing

QS is a process of chemical communication between bacterial species that relies on the production, release, detection, and population-level response to extracellular signaling molecules (known as autoinducer), enabling individual bacteria to acquire information about their changing environmental conditions. Then, they synchronously change their behavior to cope with phenotypic changes in population density and species composition in neighboring communities, and coordinating their activities with their neighboring strains (Moreno-Gámez 2023). Currently, QS-mediated communication is widely accepted in the bacterial world, and the signal-inducing factors of QS have been extensively explored (Schikora et al. 2016, Zhao et al. 2020, Mukherjee and Bassler 2019). Recent findings showed that QS is widespread in the microbial world including bacteria, fungi, and virus, and expanded our understanding of QS-mediated communication networks (Whiteley et al. 2017, Tian et al. 2021). The cell density-dependent signaling system allows microbial cells to act as multicellular organisms in response to environmental cues during various microbial behaviors, such as morphogenesis, pathogenesis, mutualistic coordination, and competition, which are used as a survival strategy in natural environments (Chen et al. 2022). Chen et al. (2023) discovered that the secreted signaling molecules, *N*-butyryl-l-homoserine lactone (C4-HSL), were closely related to salinity, and the unclassified_f__Enterobacteriaceae and Clostridium_sensu_stricto_1, associated with QS genes and butyrate production, were positively associated with C4-HSL by microbial community analysis. However, there are few studies about the QS between PGPR population, PGPR and fungi for promoting plant salt tolerance and growth in saline-alkali soil, which will be a focus for the future.

## Future prospects

HT-PGPR have drawn considerable attention for their surprising potential in agricultural production. But there are also many problems:

1. Under salt stress, a number of salt-tolerant genes, hormones, etc. involved in the salt-tolerance signaling network of HT-PGPR and their interactions are not yet clear, and the target sites of these signaling pathways remain unknown. Furthermore, the salt tolerance of the strain is controlled by multiple genes; therefore, functional studies on a single gene are not feasible.
2. Despite the fact that numerous gene mutations have been reported to affect the composition of plant microbiome under saline conditions, it remains challenging to establish the definitive causal relationship between changes in specific microbial communities and plant phenotypic output, such as improved environmental fitness of microbiota centered on HT-PGPR, due to the complexity of microbiome, and the rules of microbiotic community assemblage are not yet fully understood. This further limits our comprehension of the conundrum of a plant’s first genome working in tandem with its second genome (the microbiome) to improve nutrient efficiency.
3. Because large differences exist in indigenous microbial communities and soil heterogeneity in different saline regions, the survival and competition, colonization patterns, and interactions with plant root exudates of single or multiple beneficial strains directly applied in the rhizosphere are uncertain. It is hard to ensure that inoculant products always play targeted effects (promoting growth, increasing yield, defensing nematodes, preventing root rot, etc.). Furthermore, maintaining a similar efficiency of HT-PGPR in the agricultural sector as that under controlled laboratory conditions is another key field that must be addressed for achieving sustainable agricultural production on saline land. Also, some Sebacinales have related bacteria, which together act effectively to support plant performance under salt stress; nevertheless, it is not well elaborated how HT-PGPR and fungal interactions contribute to alleviating plant salt stress.

Elucidating the precise molecular mechanisms of HT-PGPR stress responses under saline conditions and their role in the signaling pathway via integrating interdisciplinary ideas and technologies—these will open a new avenue for capitalizing on the cultivable microorganism to strengthen plant salt tolerance and thus to refine agricultural practices and production under saline conditions. Exploring the interaction mechanism between HT-PGPR and other microorganisms, and developing new combinatorial technologies based on their interactions to achieve the “superposition or complementarity” of functional strains in plant growth and stress resistance—these will strive to solve the problem that traditional single process research cannot systematically improve the productivity of saline–alkali land, and opening up a cross-innovation field of plant-soil-microbial interaction and efficiency improvement. Excavating the genetic and molecular mechanisms of plant-mediated dynamic regulation of their own microbiota under salt stress at different analytical levels, for example, experiments from transcript analysis of both partners down to the metabolites involved at both sides could be carried out, thereby elucidating the cascaded amplification mechanism from microbiota interactions to core microbiome and plant interactions, and elevating plant–microbial interactions from qualitative description to functional analysis and systematic quantitative level—these will support the development of stress-resistant plants that can recruit beneficial microbiota in a timely and robust manner to alleviate stress. Based on the conceptual innovation of “holobiont,” the bottleneck problem that restricts the productivity of saline–alkali land will be addressed from three perspectives: plant genotype, rhizosphere core microorganism, and rhizosphere microecological orientation regulation. These will shed light on the structure, function, and role of microbial consortia in the mobilization, absorption, and utilization of nutrients and also establish a comprehensive management strategy to boost the biological potential of the plant holobiont through multi-interface interactions, resulting in a breakthrough in the cross-innovation field of plant-saline soil and salt-tolerant microorganisms.

## Conclusion

HT-PGPR are an excellent green alternative that facilitates plants to cope with increasing salinity stress, thereby addressing the global challenges of agricultural sustainability and food security posed by population growth and environmental pressures. This article expatiates the current situation of global soil salinization. Moreover, the role of HT-PGPR in plant salt stress is summarized, mainly involving the physiological and molecular mechanisms of regulating plant salt tolerance signaling pathways. Utilization of omics tools to explore the strategy of salt-stressed plants to address their “cry for help” to microorganisms by reshaping their microbiota is elaborated. We propose that the whole native soil microbiome centered on HT-PGPR should be regarded as a functional unit, while emphasizing the application of SynComs composed of HT-PGPR to promote sustainable solutions to salinization problems.

## Data Availability

All relevant data are included in the main text.
